# Proteolytic Activity Inhibition in Gingival Fluid by Cysteine Protease Inhibitors Obtained from Egg White and *Fallopia japonica* Extract: An In Vitro Study

**DOI:** 10.3390/biomedicines13071545

**Published:** 2025-06-25

**Authors:** Maciej Siewiński, Maciej Dobrzyński, Krzysztof Gołąb, Maciej Janeczek, Łukasz Nieradko, Barbara Bażanów, Andrzej Rapak, Marius Boariu, Stefan-Ioan Stratul, Alla Belova, Sorina Mihaela Solomon, Renata Samulak, Monika Machoy

**Affiliations:** 1 Institute of Inhibitory Therapy Foundation, ul. Rudolfa Weigla 12, 53-114 Wrocław, Poland; ms@therapyraft.com; 2 Department of Pediatric Dentistry and Preclinical Dentistry, Wroclaw Medical University, 50-367 Wroclaw, Poland; maciej.dobrzynski@umw.edu.pl; 3 Department of Pharmaceutical Biochemistry, Wroclaw Medical University, 50-367 Wrocław, Poland; krzysztof.golab@umw.edu.pl; 4 Department of Biostructure and Animal Physiology, Faculty of Veterinary Medicine, Wrocław University of Environmental and Life Sciences, 50-375 Wrocław, Poland; maciej.janeczek@upwr.edu.pl; 5 TherapyRaft llc, 51-678 Wroclaw, Poland; l.n@therapyraft.com (Ł.N.); andrzej.rapak@hirszfeld.pl (A.R.); 6 Department of Pathology, Faculty of Veterinary Medicine, Wrocław University of Environmental and Life Sciences, 50-375 Wrocław, Poland; barbara.bazanow@upwr.edu.pl; 7 Department of Experimental Oncology, Ludwik Hirszfeld Institute of Immunology and Experimental Therapy, 53-114 Wroclaw, Poland; 8 Department of Endodontics, Faculty of Dental Medicine, TADERP Research Center, “Victor Babes” University of Medicine and Pharmacy, 300041 Timisoara, Romania; 9 Department of Periodontology, Faculty of Dental Medicine, Anton Sculean Research Center for Periodontal and Peri-Implant Diseases, “Victor Babes” University of Medicine and Pharmacy, 300041 Timisoara, Romania; stratul.stefan@umft.ro (S.-I.S.); alla.belova@umft.ro (A.B.); 10 Department of Periodontology, Faculty of Dental Medicine, Gr. T. Popa University of Medicine and Pharmacy, 700115 Iasi, Romania; sorina.solomon@umfiasi.ro; 11 Department of Periodontology, Pomeranian Medical University, 70-204 Szczecin, Poland; renata.samulak@pum.edu.pl (R.S.); monika.machoy@pum.edu.pl (M.M.)

**Keywords:** cysteine peptidases, cystatins, periodontitis

## Abstract

**Background/Objectives**: Gingipains produced by *P. gingivalis* have been shown to be directly related to periodontal tissue degradation and are significant molecular targets in therapy of periodontitis. Blocking the activity of these enzymes should reduce survival of this pathogen and mitigate the effects of inflammation in periodontitis. Therefore, gingipains inhibitors and specific antibodies could be recommended in the treatment of periodontitis. Cysteine peptidase inhibitors can be obtained by chemical synthesis, or isolated from natural raw materials. This research has the following aims: 1. to analyze in vitro the inhibition of cysteine protease activity in the gingival crevicular fluid (GCF) and 2. to compare the toxicity of natural raw inhibitors (obtained from *Fallopia japonica* plant and egg white) with chlorhexidine (CHX) using an MTS viability test. **Methods**: Samples of GCF were collected from healthy (N = 17) individuals and (N = 65) periodontal patients. Cysteine peptidase activity was inhibited by adding a solution of cystatin from egg white (with 20% glycerol), or cystatin from knotweed, or low molecular weight inhibitors (MW < 3 kDa) from egg white and knotweed against *N*α-Benzoyl-DL-arginine 4-nitroanilide hydrochloride. **Results:** There was a statistically significant difference between the inhibition means of cysteine protease activity for the five groups (*p* < 0.001). Means for the four groups of patients with periodontitis were not statistically significant different from each other (*p* = 0.320). The inhibition rates were higher in periodontitis patients. The toxicity of knotweed cystatin inhibitor was several times lower than the toxicity of E-64d, and of CHX. **Conclusion:** Cysteine protease inhibitors isolated from egg or plants were non-toxic, effectively inhibited the activity of cysteine proteases in GCF, and may be a promising alternative to more toxic standard antimicrobials (CHX) in preventing periodontal tissue breakdown.

## 1. Introduction

The oral cavity is an open microecological environment with more than 700 species of microbes that, under normal conditions, maintain the dynamic stability of the host’s immune system [1]. When the balance between bacteria is disturbed, opportunistic pathogens begin to dominate, including major contributors to periodontitis. The element initiating the entire immune-inflammatory response responsible for the destruction of periodontal tissues are Gram-negative bacteria, mainly *Porphyromonas gingivalis*, *Treponema denticola*, *Tanerella forsythia*, and *Aggregatibacter actinomycetemcomitans*. These bacteria and their toxins stimulate immunologically competent cells to secrete inflammatory mediators (mainly enzymes), which in turn are responsible for the local destruction of teeth-supporting structures, i.e., connective and bone tissue [2]. The group of proteolytic enzymes produced in *P. gingivalis* inhabiting the periodontal pocket is known as gingipains (R1, R2, and K). They have also been shown to be directly related to periodontal tissue degradation and have become significant molecular targets in therapeutic approaches of periodontal disease, accounting for about 85% of all extracellular proteolytic activity in the periodontal pocket. It is assumed that blocking the activity of these enzymes should both reduce the survival of this pathogen and mitigate the effects of periodontitis and systemic disorders [3,4]. Therefore, gingipains inhibitors and specific antibodies could be recommended in the treatment of periodontitis [5]. Cysteine peptidase inhibitors can be obtained by chemical synthesis, or isolated from natural raw materials. The former are mainly studied in vitro and exceptionally in vivo in animal models, and are most often excluded from further studies due to their toxicity [6]. However, some of the synthetic ones, like chlorhexidine (CHX), are currently widely used, with CHX being considered the most efficient antimicrobial for the treatment of periodontitis. Its effectiveness was considered more important than its side effects. It is mostly used as an ingredient in toothpastes, gels, or mouthwashes in patients with inflammatory changes in the gums [7]. However, CHX solutions raised over the time several concerns regarding adverse drug reactions (ADR), which may include taste alteration, numbness in mouth and tongue, pain in mouth and tongue, xerostomia, and subjective discoloration [8,9,10]. Although the “loss of taste” and “numb feeling” were significantly more frequent with the 0.12% and 0.2% concentrations compared to 0.06%, severe ADR including erosion and ulceration of oral mucosa were not reported [9,10]. Some of the most frequent ADR of CHX mouthwash and gel also included xerostomia, hypogeusia, and a discoloration of the tongue; as well as calculus and extrinsic tooth staining in long-term use [11,12]. Less common ADR include swelling of the parotid gland, oral paraesthesia, glossodynia, and desquamation of the oral mucosa [11]. However tooth staining is the number one ADR that discourages patients from using chlorhexidine [11].

In the treatment of periodontitis, in addition to CHX, antibiotics from the tetracycline family, such as doxycycline and its derivatives, are also administered. They act as inhibitors of enzymes, including metalloproteinases. Tetracyclines at a concentration of 100 μM completely inhibit the amidolytic activity of arginine-specific gingipain (HRgpA and RgpB), while being less effective for the inhibition of Kgp (a Lys-x-specific protease), thus being recommended for patients with advanced periodontitis and deep periodontal pockets. A chemical modification of tetracyclines was also introduced to limit their antibiotic activity while maintaining and even enhancing their inhibitory, anti-collagenase properties in the so-called host modulating therapies of periodontitis [13]. However, as with any other antibiotics, tetracyclines raise the persistent problem of creation of antibiotic resistance, particularly in systemic administration [14]. Another example of molecules capable of blocking gingipain activity are inhibitors obtained by chemical synthesis, like KYT-36 and KYT-41. They are known to selectively inhibit gingipains. It has been suggested to administer them topically, as components of new anti-inflammatory drugs [15,16].

In contrast with the aforementioned synthetic molecules, extracts of plant and other natural preparations (i.e., from rice, cranberries, or casein after digestion by rennet) are also used, with gingipain-inhibiting properties. They are non-toxic, seem to possess the same effectiveness as CHX or tetracyclines, and it is assumed that they could be used in the prevention and treatment of periodontitis. To date, natural cystatins obtained from human saliva (S, SN, C), egg white, and citrus have also been studied on a laboratory scale, showing that cystatin S and its isolated analog from eggs block even the development of *P. gingivalis* [17,18]. Our team previously proposed the use of natural cysteine inhibitors—peptidases obtained from natural raw materials, such as egg whites and *Fallopia japonica*, which are stable and non-toxic, and were isolated based on own patents [19,20]. It was suggested that they can be applied locally on gingival tissues or on the entire oral cavity, in the form of water aerosols (e.g., in nebulizers or as spray in any concentrations), or as orodispersible tablets [19]. Despite the availability of synthetic and natural gingipain inhibitors, there are few studies directly comparing their efficacy and safety in periodontal patients. For instance, while the ability of cystatins to inhibit the growth of *P. gingivalis* was demonstrated, the mechanisms of this inhibition remain unclear [17].

Potempa et al. investigated the selective inhibition of gingipains by KYT-36 and KYT-41, highlighting their potential for localized application but noting their systemic toxicity [4]. The role of protease inhibitors mitigating periodontal destruction was explored, though primarily focused on either natural or synthetic agents rather than direct comparisons [2,21]. Specifically, the combined analysis of natural inhibitors, such as cystatins obtained from *Fallopia japonica* and egg whites, and of widely used synthetic agents, including CHX remains underexplored [13]. Cystatins and polyphenols from *Fallopia japonica* address multiple aspects of periodontal disease. Cystatins inhibit gingipains while sparing host proteases [22], and polyphenols help reduce biofilm formation and inflammation, promoting periodontal health [4,14]. Furthermore, the integration of these natural agents into multimodal treatment strategies recommended by the EFP clinical practice guidelines, which emphasize biofilm control, inflammation reduction, and host modulation, has not been sufficiently studied [4,14,17].

This research presents the following aims: 1. to analyze in vitro the inhibition of cysteine protease activity in the gingival crevicular fluid (GCF) and 2. to compare the toxicity of natural raw inhibitors (obtained from *Fallopia japonica* plant and egg white) with chlorhexidine (CHX). The study hypothesis is that the natural inhibitors of cystein protease are less toxic and more efficient than CHX used in controlling periodontal inflammation.

## 2. Materials and Methods

### 2.1. Ethical Considerations

The study was conducted according to the guidelines of the Declaration of Helsinki and approved by the Bioethics Committee of Medical University of Wroclaw KB no 210/2002. The participants were provided with the necessary information regarding the purpose of the study and the experimental design. Informed consent was obtained from all subjects involved in the study.

### 2.2. Study Population

**Figure 1:**
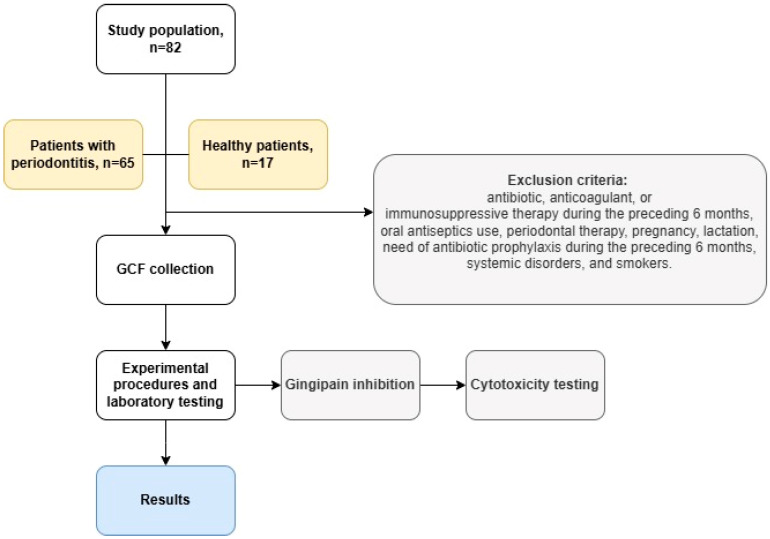
Flowchart of the study.

The study included 82 individuals aged 35–60 years, of both genders, divided into two groups. The first group (n = 65) included patients with a clinical diagnosis of periodontitis, at all stages and grades, according to the classification of 2018 [19]. The second group (n = 17) consisted of subjects with clinically healthy periodontium (periodontal pockets ≤ 3 mm and less than 10% bleeding on probing) [19]. Participants met the following inclusion criteria: diagnosis of periodontitis or periodontal health, age > 18 years, and no contraindications for periodontal treatment or research. Criteria for exclusion included the following: patients who underwent antibiotic, anticoagulant, or immunosuppressive therapy during the preceding 6 months, patients who used oral antiseptics or received any periodontal therapy, pregnant or lactating women, patients in need of antibiotic prophylaxis during the preceding six months, systemic disorders, and smokers. The study flowchart is represented in Figure 1.

### 2.3. Sourcing and Quality Control of Raw Materials

*Fallopia japonica* plants were purchased from an authorized pharmaceutical company and were identified in the Department of Pharmaceutical Biochemistry, Wroclaw Medical University. Unfertilized eggs were obtained from the chicken pen at the Faculty of Veterinary Medicine of the Wrocław University of Environmental and Life Sciences on the day of laying and stored at 4 °C for up to 3 days.

### 2.4. Chemical Reagents

The following reagents were used in the present experiments: BApNA (*N*α-Benzoyl-DL-arginine 4-nitroanilide hydrochloride, CAS 911-77-3), Fast Garnet GBC (CAS 97-56-3), Chlorhexidine digluconate solution (20–25%), E-64d protease inhibitor (Aloxistatin, CAS 88321-09-9), papain (CAS 9001-73-4), and Sepharose 4B^TM^ (CAS 9012-36-6), all from Sigma-Aldrich (Steinheim, Germany). The remaining chemically pure reagents were produced by POCH (Gliwice, Poland).

### 2.5. Biological Material

Filter paper strips (3 mm strips of Whatman tissue paper grade 3, Sigma-Aldrich) were used to collect GCF by inserting them into the sulcus in apical direction, until mild resistance was detected, or by inserting the strips at or over the entrance of the pocket to pick up the seeping fluid [20]. The strips were left for 30 s, removed, and inserted in transportation tubes with 2 mL PBS and were immediately stored at −80 °C [23].

The activity of cysteine gingipains was inhibited by low molecular weight (<3 kDa) cysteine peptidase inhibitors and cystatins isolated from dry powdered egg white or knotweed homogenate (*Fallopia japonica*). Cystatin obtained from egg white required the addition of 20% glycerol to maintain stability [20].

### 2.6. Step-by-Step Protocol for Isolating Cysteine Peptidase Inhibitors

#### 2.6.1. Reagent Preparation

The following protocol [20] describes the isolation of cysteine peptidase inhibitors with electrophoretic purity from biological sources. Biological materials, such as egg proteins, plant homogenates (e.g., *Fallopia japonica*), or mammalian tissues (e.g., placentae or amniotic fluid), are diluted in distilled water in a 1:1 ratio to create a homogeneous protein solution. The potential of hydrogen (pH) is adjusted to 2.0 using 0.1 molar (M) hydrochloric acid (HCl), and the solution is heated to 90 degrees Celsius (°C) for 10 to 20 min in a temperature-controlled water bath to denature impurities while preserving inhibitor activity. The mixture is then cooled to room temperature (22 °C), neutralized with 0.1 M sodium hydroxide (NaOH) until it reaches a pH of 7.0, and frozen at −20 °C for at least 12 h to promote the separation of unwanted proteins.

#### 2.6.2. Inhibitor Isolation and Purification

After freezing, the solution containing the inhibitor is completely thawed at room temperature (22 °C) and centrifuged at 30,000 revolutions per minute (RPM) for 20 min at 4 °C in a refrigerated high-speed centrifuge. The supernatant, which contains enriched cysteine peptidase inhibitors, is carefully collected and loaded onto a chromatography column packed with Sepharose 4B resin bound to immobilized papain or phycin. The column is pre-equilibrated with phosphate-buffered saline (PBS) at pH 7.4 to optimize binding.

Elution of inhibitors is performed using carbonate buffers adjusted to pH 9.5, 11.0, or 12.0, with the addition of 20% glycerol to stabilize the inhibitors and prevent aggregation. The process is carried out at controlled temperatures ranging from 8 °C to 55 °C, depending on the desired purity and inhibitor activity. Protein-containing fractions are collected and acidified to pH 2.0 with 0.1 M HCl, briefly reheated to 90 °C for 10 to 20 min, cooled, and neutralized with 0.1 M NaOH to a final pH of 7.0 to ensure stability. To concentrate the inhibitors and remove low-molecular-weight contaminants, ultrafiltration using membranes with a molecular weight cutoff (MWCO) of 5 kilodaltons (kDa) is employed.

Chromatography gel can be reused multiple times. After elution, the gel is washed with 0.2 M sodium chloride (NaCl) and distilled water to remove unbound proteins, followed by reactivation in phosphate buffer (pH 6.5) containing 2 millimolar (mM) dithiothreitol (DTT), 2 mM ethylenediaminetetraacetic acid (EDTA), and 0.05% mercaptoethanol at 4 °C for at least 12 h. For single-use applications, inhibitors can alternatively be eluted at higher temperatures ranging from 60 °C to 100 °C to maximize efficiency.

#### 2.6.3. Testing the Activity of Purified Inhibitors

Purified inhibitors are stored at 4 °C in sterile ampoules for short-term use or frozen at −80 °C in solutions containing 20% glycerol for long-term preservation. To confirm inhibitor activity, spectrofluorometric assays are performed using a fluorogenic substrate specific for cysteine proteases, such as Z-Phenylalanyl-Arginine-7-amino-4-methylcoumarin (Z-Phe-Arg-AMC). The fluorescence is measured using a microplate reader (SpectraMax M5, Marshall Scientific, Hamptin, NH, USA) with excitation and emission wavelengths set at 380 nanometers (nm) and 460 nm, respectively. Additional validation is performed using colorimetric assays with N-benzoyl-DL-arginine-p-nitroanilide (BAPNA) as the substrate, with absorbance measured at 405 nm using a spectrophotometer (Fluorescence Spectrophotometer SP-FL96, Scitek Global Co., Ltd., Jinan, China).

### 2.7. Experimental Procedures

#### 2.7.1. Inhibition of Cysteine Gingipain Activity Against BApNA

A 2.0 mL PBS sample with a strip of Whatman 3 tissue paper soaked in gingival crevicular fluid GCF was sonicated for 30 s on ice and then centrifuged at 10,000 rpm for 10 min. The cysteine peptidase activity was inhibited by adding a solution of cystatin from egg white (with 20% glycerol) or cystatin from knotweed or low molecular weight inhibitors (MW < 3 kDa) from egg white and knotweed against BApNA [24,25].

#### 2.7.2. Measurement of Cytotoxicity of Inhibitors

The aim of the measurement was to assess the effect of the test substances on the survival of peripheral blood mononuclear cells (PBMCs) isolated from human blood. Data were standardized for a control sample. The toxicity of isolated cystatins and cysteine peptidases with a low molecular weight of <3 kDa from egg white and knotweed was compared with chlorhexidine and E-64d using an MTS viability test [12,26]. All inhibitors were used in the concentration range of 0.01 to 100 micrograms/mL.

### 2.8. Statistical Analysis

The sample size was determined based on statistical power analysis, ensuring sufficient sensitivity to detect differences in inhibitor efficacy. A power calculation with 80% power (β = 0.20) and a familywise significance level of 0.05 (α = 0.05) indicated that 16 participants per group were needed to detect an effect size of 1.2 (i.e., a mean difference between groups of interest equal to 1.2 standard deviations) when comparing the healthy group with each one of the four patient groups.

Statistical analysis was performed in GraphPad Prism version 9.51. One-way analysis of variance (ANOVA) was used to assess the impact of the tested inhibitors on the proteolytic activity of the analyzed samples. D’Agostino–Pearson tests were used to evaluate the normal distribution assumption for each one of the five groups of interest. There were no indications of violation of the normality assumption (all *p* > 0.10). We performed the Brown–Forsythe test of homoscedasticity. Because the equal variance assumption was rejected (*p* < 0.001), we used Welch’s ANOVA, a version of ANOVA that allows for unequal variances. Since the research aim was to compare the four patient groups with the healthy group, i.e., 4 pairwise comparisons of interest, we used Dunnett’s method to adjust for multiple testing and control the familywise type I error at the 0.05 significance level.

For cell viability, we only calculated descriptive statistics and plotted them. All results were presented as arithmetic mean and standard deviation.

## 3. Results

**Figure 2:**
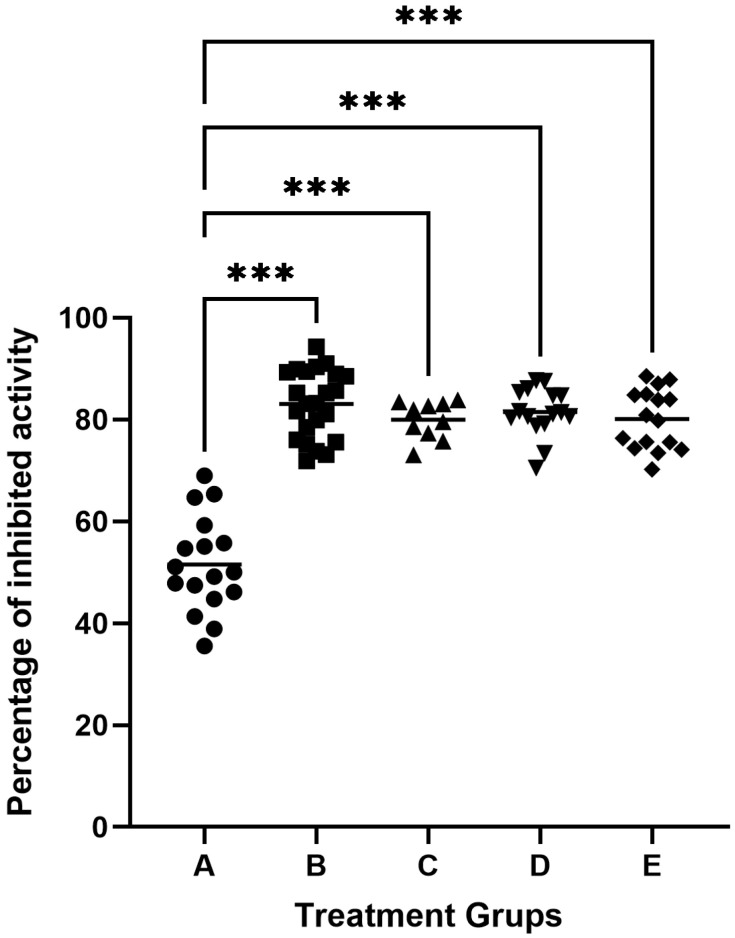
Inhibition of cysteine protease activity in the gingival crevicular fluid (GCF). A—inhibitory activity of egg white cystatin in patients with healthy periodontium (n = 17); B—inhibitory activity of egg white cystatin in patients with periodontitis (n = 23); C—inhibitory activity of low-molecular egg white inhibitors in patients with periodontitis (n = 10); D—inhibitory activity of cystatin from knotweed in patients with periodontitis (n = 16); and E—inhibitory activity of low-molecular knotweed inhibitors in patients with periodontitis (n = 16). *** *p* ≤ 0.001.

The study used 82 samples of gingival crevicular fluid (GCF). One sample each from a healthy person and a patient with periodontitis were used to inhibit gingipain activity in 100 μL GCF. The samples were incubated with inhibitor solutions of 50 μL, 100 μL, 200 μL, or 500 μL (cysteine or low) derived from egg white or knotweed. We found that the maximum degree of inhibition of gingipain was obtained with the addition of 100 μL inhibitors with an activity of 5.0 units/mL, which accounted for about 85% of proteolytic inhibition in GCF. The GCF activity of subjects classified as healthy was 51.54 ± 9.36% inhibited by egg protein cystatins. In contrast, in patients with periodontitis, it was found that the degree of proteolytic inhibition by egg protein cystatin was 83.08 ± 6.63% of primary activity in GCF (Figure 2). Adjusting for multiple testing by using Dunnett’s method, there were statistically significant differences between the means of the inhibition of cysteine protease activity of each one of the four patient groups and the mean of the inhibition of cysteine protease activity of the healthy group (all adjusted *p* < 0.001), see Figure 2. The analysis of inhibition rates revealed marked differences between healthy individuals and periodontitis patients, as reflected in the boxplot (Figure 2). In healthy individuals (Group A: healthy controls), the mean inhibition rate was 48.16% ± 8.94%, with values ranging from 31.03% to 68.97%. In contrast, periodontitis patients demonstrated higher and more consistent inhibition rates across treatment groups. For example, in Group B, for periodontitis patients treated with egg white-derived cystatin, the mean inhibition rate was 80.10% ± 5.89%, while in Group C periodontitis patients treated with low-molecular-weight cystatin achieved a mean inhibition rate of 83.08% ± 6.63%, with inhibition rates ranging from 71.89% to 94.33%. Similarly, for Group D, periodontitis patients treated with knotweed-derived cystatin achieved a mean inhibition rate of 81.48% ± 5.89%, reflecting the efficacy of natural inhibitors in advanced periodontitis. Similar results were obtained by inhibiting cysteine gingipains in GCF samples from patients with periodontitis, using low molecular weight inhibitors from egg white, knotweed cystatin, and low-molecular knotweed inhibitors (Figure 2).

**Figure 3:**
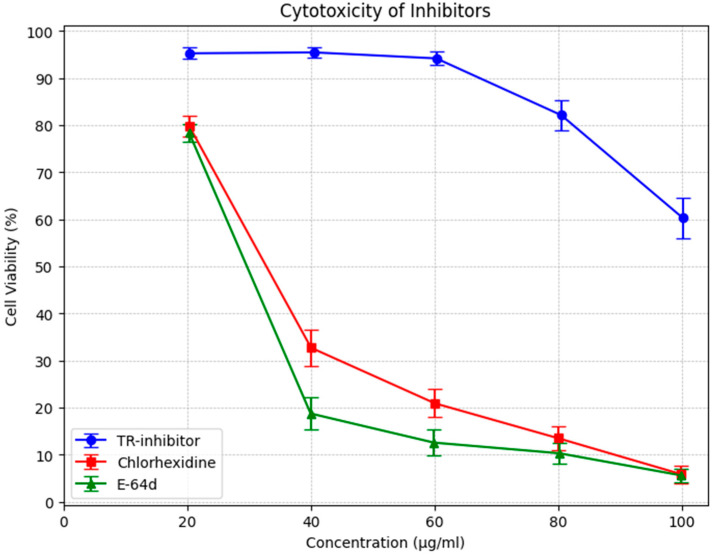
MTS test with human PBMC. Comparison of the toxicity of cysteine protease inhibitors (TR) isolated from knotweed with chlorhexidine and E 64d. Data were presented as arithmetic mean and standard deviation.

In addition, knotweed cystatin (papain-like cysteine proteinase inhibitor, TR-inhibitor) was shown to have very low toxicity as measured by peripheral blood mononuclear cell survival (PBMC). The results of the study demonstrated that the toxicity of TR-inhibitor is several times lower than the toxicity of E-64d, a commonly used cysteine protease inhibitor, and of chlorhexidine, which is a component of many commercially available preparations recommended for the treatment of periodontitis (Figure 3).

The cytotoxicity assessment of TR-inhibitor, chlorhexidine (CHX), and E-64d revealed notable differences in cell viability across the tested concentrations.

TR-inhibitor displayed minimal cytotoxicity, with cell viability consistently above 94% at lower concentrations and remaining above 60% even at the highest dose (100 µg/mL). Although a slight dose-dependent decline was observed, the overall stability suggests that TR-inhibitor is well-tolerated and suitable for prolonged biological applications. Statistical analysis confirmed this, with a mean viability of 85.04% (95% CI: 74.52–95.56%).

In contrast, CHX demonstrated a pronounced cytotoxic effect, with viability dropping sharply beyond 40 µg/mL. At 20 µg/mL, cell viability was 79.85%, but by 100 µg/mL, it had decreased to just 5.88%. The broad confidence interval (31.66%, 95% CI: 10.89–52.43%) reflects greater variability, especially at higher doses.

E-64d exhibited a similar cytotoxic pattern to CHX, with a rapid decline in cell viability as concentration increased. At 20 µg/mL, viability was 78.38%, closely resembling CHX at the same dose. However, at 100 µg/mL, viability had plummeted to 5.64%. The confidence interval (24.31%, 95% CI: 7.25–41.37%) further supports its high toxicity at elevated concentrations.

## 4. Discussion

Problems associated with periodontitis pose a significant therapeutic challenge, not only due to localized tissue destruction but also because of its links to various systemic diseases, including cardiovascular disorders, neurodegenerative diseases, cancer, and gynecological complications [27,28]. The primary pathogenic driver of these processes is *Porphyromonas gingivalis*, whose most virulent factors are exogenous proteolytic enzymes known as gingipains. These enzymes account for up to 85% of the total proteolytic activity in periodontal pockets, playing a key role in tissue degradation and systemic complication [22,24].

To date, the most effective antimicrobial agents in periodontitis treatment have been chlorhexidine (CHX) and tetracycline-group antibiotics. However, concerns over cytotoxicity, adverse effects, and antimicrobial resistance have led researchers to investigate alternative approaches [12,24]. While synthetic inhibitors such as KYT-36 and E-64 have demonstrated selective gingipain inhibition, their systemic toxicity and unintended effects on host enzymes [29,30] have limited their clinical utility. In contrast, natural inhibitors, such as cystatins derived from egg whites and plant-derived compounds from *Fallopia japonica*, have emerged as promising alternatives. These inhibitors selectively target gingipains, reducing their proteolytic activity while maintaining a favorable safety profile. Studies have demonstrated their effectiveness in inhibiting *P. gingivalis* growth and protease activity without the cytotoxicity associated with synthetic agents [31,32].

Traditional periodontal therapies focus primarily on mechanical debridement and root surface instrumentation to maintain periodontal health and promote tissue regeneration [33,34]. However, a more targeted approach, involving selective inhibition of gingipains, has the potential to mitigate inflammatory destruction at the molecular level. Our study explored natural cysteine peptidase inhibitors (CPIs), extracted from biologically stable, non-toxic sources, as alternative agents for gingipain inhibition in periodontal therapy. The results indicate that these inhibitors effectively block 85% of the total proteolytic activity in gingival crevicular fluid (GCF), with remaining enzymatic activity likely attributed to other cysteine proteases not targeted by CPIs [3,4,33].

Interestingly, we observed variability in inhibition rates between healthy individuals and periodontitis patients, likely due to inherently lower and more heterogeneous baseline gingipain activity in healthy periodontal tissues. This variability may stem from inter-individual differences in GCF composition or inhibitor responsiveness. Statistical analysis (*p* < 0.05) confirmed that natural inhibitors exhibited stronger efficacy in periodontitis patients than in healthy individuals, further highlighting their potential for targeting gingipain activity effectively in diseased conditions [4,17]. These findings reinforce the necessity of tailoring treatments to enzymatic differences between health and disease, in line with previous research suggesting selective gingipain inhibition as a viable strategy [2,14].

Research on gingipain inhibition strategies has significant implications for periodontal treatment innovations, particularly in developing selectively acting inhibitors as alternatives to CHX [3,13,30,35].

According to the “docking model” for the interaction of cystatins with cystein proteinases based on the x-ray crystal structure of the Gly-form of egg-white cystatin [36], three parts of the inhibitor are in close contact with the active site cleft of papain: the amino terminus; a first hairpin loop (residues 53–57) containing the egg white cystatin QLVSG variation in the prototype sequence QVVAG, conserved in almost all members of the cystatin superfamily; and a second hairpin loop (residues 102–107) containing the conserved Trp-104. The Leu-8 of chicken cystatin seems to interact specifically with the Sz-subsite of papain.

Our research group previously patented a proprietary method for isolating natural cysteine peptidase inhibitors, which include cystatins (~13 kDa molecular weight) and low-molecular-weight inhibitors (<3 kDa) [20]. The delivery of these inhibitors as an aqueous aerosol via nebulization may further enhance their clinical applicability, ensuring broader oral cavity distribution beyond conventional antimicrobial mouth rinses and pastes.

A key finding of our study was the exceptionally low toxicity profile of CPIs isolated from *Fallopia japonica* when compared to CHX—the current gold standard antimicrobial in periodontitis [12]. While CHX formulations (mouthwashes, gels, and pastes) are used at concentrations between 0.12 and 0.20% to balance antimicrobial activity with cytotoxicity, our CPIs demonstrated high efficacy without such limitations. In our toxicity assays using peripheral blood mononuclear cells (PBMCs), CPIs showed significantly higher biocompatibility, reinforcing their potential as a safer alternative.

Moreover, natural CPIs could provide a solution to the growing problem of antibiotic resistance in periodontal pathogens. Long-term use of tetracyclines has been associated with the emergence of antibiotic-resistant bacterial strains, making the need for alternative therapies increasingly urgent [37]. By targeting gingipain activity without inducing bacterial resistance, natural CPIs may serve as an effective adjunct or replacement for antibiotics in periodontal therapy.

Previous studies analyzing GCF samples from patients with advanced periodontitis have reported 85% inhibition of gingipains, with 15% of enzymatic activity remaining unaffected [21]. This suggests that while CPIs significantly reduce gingipain activity, complete inhibition may require combination therapies or higher inhibitor concentrations. Blocking gingipain activity weakens *P. gingivalis* virulence, making it more susceptible to host immune defenses and bacterial clearance mechanisms. Furthermore, previous studies have demonstrated that natural cysteine peptidase inhibitors—such as egg white cystatin, cystatin C, and salivary cystatin S—effectively inhibit *P. gingivalis* growth in vitro [15,18].

A critical factor when evaluating cysteine peptidase inhibitor (CPI) sources is their structural stability and feasibility for large-scale production. While human cystatin C has been extensively studied, its recombinant forms often exhibit dimerization, which can compromise bioactivity and complicate purification at an industrial scale [38]. In contrast, CPIs derived from egg white and *Fallopia japonica* offer greater stability and ease of extraction, making them more practical for periodontal applications. This distinction is particularly relevant given the increasing need for cost-effective, scalable alternatives to synthetic inhibitors.

Our method for isolating and purifying cystatin inhibitors from natural sources aligns with prior research on plant-based recombinant protein expression [39]. Systems such as *Fallopia japonica* enable stable, large-scale production of cystatins while preserving their inhibitory activity, providing a viable alternative to recombinant cystatins from egg white or human cystatin C, which often suffer from loss of function due to dimerization [19,38,40,41,42]. The ability to harness plant-derived CPIs for therapeutic applications represents a promising avenue for the development of new, stable, and non-toxic alternatives for periodontal treatment.

One limitation of our study was the relatively small sample size, which prevented further stratification based on disease severity. Future studies should explore CPIs in larger patient cohorts and include in vivo models to validate their efficacy in clinical settings. Once therapeutic molecules gain regulatory approval, clinical trials assessing their anti-inflammatory and antibacterial properties in periodontitis will be a critical next step.

## 5. Conclusions

Within the limits of the current study, it was found that cysteine protease inhibitors isolated from egg or plants were non-toxic and effectively inhibited the activity of cysteine proteases in GCF and may be a promising alternative to much more toxic standard antimicrobials like Chlorhexidine in preventing periodontal tissue breakdown. Future research should elucidate the best delivery form, the most efficient method of administration, and the possibility of integrating this new category of drugs into current accepted periodontal treatment protocols.

## Data Availability

The data used to support the finding of this study are included within the article.
